# Dementia Care Coaching: A Pilot to Evaluate Acceptability and Feasibility in Care Communities

**DOI:** 10.1093/geroni/igab046.3093

**Published:** 2021-12-17

**Authors:** Lorna Prophater, Boeun Kim, Basia Belza, Sarah Cameron, Sam Fazio

**Affiliations:** 1 Alzheimer's Association, Chicago, Illinois, United States; 2 University of Washington, Seattle, Washington, United States; 3 Alzheimer's Association, Datyon, Ohio, United States

## Abstract

The Alzheimer’s Association (AA) Dementia Care Practice Recommendations (DCPR) outline ten recommendations to achieve quality care with a person-centered focus. The AA has developed tools to assist care communities (CC) to evaluate their status within the recommendations by working with a trained coach to maximize adoption and implementation of these recommendations. The purpose of this pilot was to evaluate the acceptability and feasibility of pairing trained DCPR coaches with CC teams to implement the DCPR tools. Seven CCs were recruited and four received the DCPR overview and self-assessment. Of the four CC, one withdrew and did not receive the intervention. The remaining three were located in a suburban area, nonprofit, and with memory care units. Data was collected from November 2019 through March 2020. Nine CC staff participated with a mean age 35.8 years and had worked for 11.8 years. Baseline mean scores on the Organizational Readiness to Implementing Change (ORIC) scale were 4.6 for the commitment domain and 4.4 for the efficacy domain. Mean scores on the Nursing Home Employee Satisfaction Survey were high. Sixty-nine percent of CC participants were satisfied with their jobs (greater than 4). Findings from mid-project interviews with the coaches revealed difficulty with scheduling appointments, significant efforts needed to get the “right” people at the table and need for the DCPR tools to be more user-friendly. No post-intervention results were collected due to closing of the CCs to visitors due to COVID. The DCPR tools shows promise and are being evaluated in additional CCs.

